# Characterization of a Lymphoid Organ Specific Anti-lipopolysaccharide Factor From Shrimp Reveals Structure-Activity Relationship of the LPS-Binding Domain

**DOI:** 10.3389/fimmu.2019.00872

**Published:** 2019-04-24

**Authors:** Shihao Li, Xinjia Lv, Fuhua Li, Jianhai Xiang

**Affiliations:** ^1^Key Laboratory of Experimental Marine Biology, Institute of Oceanology, Chinese Academy of Sciences, Qingdao, China; ^2^Laboratory for Marine Biology and Biotechnology, Qingdao National Laboratory for Marine Science and Technology, Qingdao, China; ^3^Center for Ocean Mega-Science, Chinese Academy of Sciences, Qingdao, China; ^4^University of Chinese Academy of Sciences, Beijing, China

**Keywords:** anti-lipopolysaccharide factor, lymphoid organ, LPS-binding domain, amino acid property, *Vibrio harveyi*

## Abstract

Anti-lipopolysaccharide factor (ALF) is a kind of important antimicrobial peptides with broad-spectrum antimicrobial activities. The LPS-binding domain (LBD) contributes to the major antimicrobial activity of ALF. However, LBDs from different ALFs share low sequence similarity. The general character of LBDs needs to be elucidated to understand the molecular mechanism of their function and facilitate LBD-original drug design. Here we identified a lymphoid organ specifically expressed *ALF*, designated as *FcALF8*, from the Chinese shrimp *Fenneropenaeus chinensis*. The synthetic LBD peptide of FcALF8 (LBD8) showed strong antibacterial activities to the pathogenic *Vibrio*, such as *Vibrio alginolyticus, Vibrio harveyi*, and *Photobacterium damselae* with a MIC value of 0.5–1, 1–2, and 1–2 μM, respectively. *FcALF8* knock-down using dsRNA led to significant increase of the viable bacteria in the lymphoid organ and hepatopancreas of shrimp upon *V. harveyi* infection. On the contrary, the proliferation of *V. harveyi* in the shrimp lymphoid organ and hepatopancreas significantly decreased after infected by LBD8 pre-incubated *V. harveyi*. Sequence alignments showed that the LBDs from 39 ALFs shared only two identical cysteine residues. However, 17 of the total 22 LBD residues showed high similarity when the amino acids were classified into hydrophobic and hydrophilic ones. A further activity analysis on modified LBD8 peptides showed that the antibacterial activity of LBD8 was lost after linearization and apparently weakened after changing the amino acid property at certain positions. The data indicated that the disulfide bond and amino acid property contributed to the conservation of the functional domain. To the best of our knowledge, this is the first identified ALFs specifically expressed in the lymphoid organ of shrimp with strong antibacterial activity. The present data will give creative instructions for the design of LBD-originated antimicrobial agents.

## Introduction

Anti-lipopolysaccharide factor (ALF) is a kind of cationic antimicrobial peptides (AMPs) with broad-spectrum activities against pathogenic microorganisms in crustacean. Since the first ALF gene was isolated from the hemocytes of the horseshoe crab *Limulus polyphemus* (1), more and more ALFs were identified in decapod crustaceans. ALFs play important roles in crustacean during innate immune responses, which are supported by abundant evidence from both the transcription and the protein levels. The transcription level of ALFs could be activated by different bacteria challenges (2–4). After knock-down of ALF genes by double-strand RNA (dsRNA), the mortality rates of shrimp infected by *Vibrio* and WSSV were apparently raised (5, 6). In the prawn *Exopalaemon carinicauda*, dsRNA-mediated knock-down of ALF gene also caused rapid proliferation of endogenous bacteria *Vibrio alginolyticus* and *Vibrio parahaemolyticus* (7). The recombinant ALF proteins exhibited *in vitro* antimicrobial activities (8) and pre-injection of recombinant ALF protein could reduce cumulative mortality of shrimp caused by *Vibrio harveyi* infection (9). In mud crab *Scylla paramamosain*, a single amino acid mutant of SpALF6 obviously changed its antimicrobial activities (10). These data revealed that ALFs were essential effectors in innate immune system of crustacean.

In general, different isoforms of ALF genes co-existed in one organism. Seven isoforms of ALF genes were isolated from *Penaeus monodon* (11, 12). Seven ALF isoforms were reported both from *Portunus trituberculatus* (4, 13–16) and *Fenneropenaeus chinensis* (17). Five ALF isoforms were identified from *E. carinicauda* (7, 18). As the functional domain of ALF, the LPS-binding domain (LBD), which is composed of alpha helixes and beta sheets (19–21), exerts the antimicrobial activities of ALF. The synthetic peptides of diverse LBDs, such as ALFSp (22), SsALF (23), and SALF_55−76_ (24), show apparent antimicrobial activities against various bacteria. The synthetic LBD corresponding to a crayfish ALF could inhibit WSSV replication in cultured Hpt cells (25). The LBDs from *F. chinensis* (26) and *E. carinicauda* (18) also show diverse anti-bacterial and anti-viral activities. These data suggest that different ALFs or LBDs have distinct antimicrobial activities. Discovery of more ALF genes will facilitate the development of antimicrobial drug and disease control.

Notably, even if different LBDs show antimicrobial activities and similar secondary structure, their sequence similarities are relatively low. In *F. chinensis*, seven reported ALFs only have three identical amino acid residues in the LBD region (26). Identification of the common features the LBD region is important for LBD-originated drug design. The physicochemical properties rather than specific amino acid residues are regarded as key factors for the antimicrobial activities of cationic antimicrobial peptides (27). As one kind of cationic AMPs, ALF might also comply with this feature. However, the common features of LBD peptides, the functional domain of ALFs, are still needed to be clarified.

In the present study, a new isoform of ALF gene (*FcALF8*), which was specifically expressed in the lymphoid organ, was isolated from the Chinese shrimp *F. chinensis*. The antibacterial function of FcALF8 was illustrated by *in vitro* detecting the inhibitory effects of the synthetic LBD peptide on several bacteria and *in vivo* analyzing the activity of *FcALF8* on *V. harveyi* infection. The LBDs of FcALF8 and other reported ALFs were compared and the similarities and differences among them were analyzed. To the best of our knowledge, *FcALF8* is the first reported ALF which is specifically expressed in the lymphoid organ of shrimp with strong antibacterial activity. The data provide new insights into the function of AMPs in crustaceans and will give creative instructions for the design of LBD-originated antimicrobial agents.

## Materials and Methods

### Animal and Tissue Collection

Adult Chinese shrimp with a body weight about 30 g were bought from a local farm. The shrimp were cultured in fiberglass tanks and fed with artificial diet for 7 days to make them acclimate to the laboratory conditions. Hemolymph was exsanguinated from the ventral sinus located at the first abdominal segment with an equal volume of anticoagulant (27 mM sodium citrate, 336 mM NaCl, 115 mM glucose, 9 mM EDTA, pH 7). Hemocytes were isolated by centrifugation at 800 g, 4°C, for 10 min, and preserved in liquid nitrogen. Meanwhile, different shrimp tissues, including lymphoid organ (Oka), gill, epidermis, muscle, hepatopancreas, intestine, ovary or testis were dissected and preserved in liquid nitrogen. These samples were used for tissue distribution analysis of target genes.

### Total RNA Extraction and cDNA Synthesis

Total RNA was extracted from different tissues with RNAiso Plus reagent (TaKaRa, Japan). The RNA quality was confirmed by electrophoresis on 1% agarose gel. About 1 μg total RNA of each sample was first treated with RQ1 RNase-Free DNase (Promega, USA) and then used to synthesize cDNAs by PrimeScript™ 1st strand cDNA Synthesis Kit (TaKaRa, Japan) with random 6 mers.

### Obtaining of FcALF8 Gene and Sequence Analysis

The cDNA sequence of FcALF8 gene was obtained from a comparative transcriptome database of the shrimp cephalothorax before and after WSSV infection (17). The DNA sequence of FcALF8 was obtained by Blastn in a genome DNA database of *F. chinensis* constructed in our lab (28) using the cDNA sequence as a query sequence. The open reading frame (ORF) of FcALF8 was confirmed by PCR with the primers FcALF8-F and FcALF8-R (Table 1) and then Sanger sequencing.

**Table 1:** Nucleotide sequences of primers used in the present study.

**Primer name**	**Sequence (5**′**-3**′**)**	**AT^**1**^ (**°**C)**
FcALF8-F	TAGGACCTGCGAGTGAAGTCAT	59
FcALF8-R	CTTCCGATTCTAATTCCCTGTG	
FcALF8-dsF	TAATACGACTCACTATAGGGTAGGACCTGCGAGTGAAGTCAT	61
FcALF8-dsR	TAATACGACTCACTATAGGGCTTCCGATTCTAATTCCCTGTG	
EGFP-dsF	TAATACGACTCACTATAGGGCAGTGCTTCAGCCGCTACCC	55
EGFP-dsR	TAATACGACTCACTATAGGGAGTTCACCTTGATGCCGTTCTT	55
FcALF8-qF	TGGACGCTTGTCTACGGCAC	59
FcALF8-qR	CAACCACGGCTTGGCATCCT	
18S-qF	TATACGCTAGTGGAGCTGGAA	56
18S-qR	GGGGAGGTAGTGACGAAAAAT	

*AT is the abbreviation of annealing temperature. T7 promoter sequences are underlined*.

The nucleotide sequence and deduced amino acid sequence of FcALF8 were analyzed using the BLAST algorithm (NCBI, http://www.ncbi.nlm.nih.gov/BLAST/). Signal peptide was predicted by CBS prediction servers (http://www.cbs.dtu.dk/services). LPS-binding domain (LBD) was predicted by SWISSMODEL (http://swissmodel.expasy.org/). Genomic organizations were carried out by the Genewise tool (http://www.ebi.ac.uk/Wise2/index.html). The other 38 ALF protein sequences from 12 crustacean species were downloaded from NCBI protein database (https://www.ncbi.nlm.nih.gov/protein). The phylogenetic tree was constructed using MEGA software version 5 (29). Alignment of LBD sequences was performed by online ClustalW2 software (http://www.ebi.ac.uk/Tools/msa/clustalw2/).

### Semi-Quantitative RT-PCR

The amount of cDNA templates for RT-PCR was quantified using 18S rRNA as an internal reference gene following the PCR condition described below: denaturation at 94°C for 2 min; 26 cycles of 94°C for 20 s, 56°C for 20 s, and 72°C for 20 s. Equal amount of cDNA from different tissues was used for detecting the expression pattern of FcALF8 transcripts following the PCR condition described below: denaturation at 94°C for 2 min; 35 cycles of 94°C for 20 s, 59°C for 20 s, and 72°C for 30 s. The primers for amplification of 18S rRNA (18S-qF and 18S-qR) and FcALF8 (FcALF8-qF and FcALF8-qR) were listed in Table 1. The PCR products were detected by electrophoresis on 2% agarose gel.

### Synthesis of LBD8 and Modified Peptides

The LBD peptide of FcALF8, designated as LBD8, was synthesized by a commercial company (Shanghai Ziyu Biotechnology Co, Ltd, China). The amino acid residues of LBD8, Ac-Yc(CSYSTRPYFIRWQLKFKTKIWC)P-NH_2_, was acetylated in the N-terminal and amidated in the C-terminal. A disulfide bond was formed between the two cysteine residues. The synthetic LBD8 peptide was confirmed by mass spectrometry and the purity was tested by high performance liquid chromatography. In order to understand the structure-activity relationship of LBD8 peptide, three modified peptides were designed, and synthesized. These modified peptides included LBD8L (Ac-YCSYSTRPYFIRWQLKFKTKIWCP-NH_2_), LBD8Q (Ac-Yc(CSYSTRPYFQRWQLKFKTKIWC)P-NH_2_), and LBD8G (Ac-Yc(CSYSTRPYFIRWQLKFKGKIWC)P-NH_2_). LBD8L did not have the disulfide bond, which was the linearized form of LBD8. LBD8Q was named after the hydrophobic residue Isoleucine at position 10 of LBD8 was replaced by a hydrophilic residue Glutamine. LBD8G was named after the hydrophilic residue Threonine at position 18 of LBD8 was replaced by a hydrophilic residue Glycine. A peptide with 24 amino acid residues, Ac-TTGKLPVPWPTLVTTFSYGVQCFS-NH_2_, based on the amino acid sequence of Green Fluorescent Protein (pGFP) (Accession number: AAN41637) was synthesized as a negative control.

### Minimal Inhibitory Concentration (MIC) Assay

The antibacterial activity of LBD8 was detected by minimal inhibitory concentration (MIC) assay. The bacteria strains used for detection contained six Gram-negative bacteria, *Vibrio alginolyticus, Vibrio harveyi, Vibrio campbellii, Photobacterium damselae, Escherichia coli* and *Vibrio parahemolyticus*, and two Gram-positive bacteria, *Micrococcus luteus* and *Staphylococcus epidermidis. V. alginolyticus, P. damselae, V. harveyi*, and *V. campbellii* were incubated in TSB growth medium overnight at 28°C. *M. luteus, E. coli, V. parahemolyticus*, and *S. epidermidis* were incubated in LB growth medium overnight at 37°C. The overnight cultured bacteria were transferred into fresh medium, incubated for 6 h. Then, 133 μL medium, 15 μL peptide solution, and 2 μL bacteria were added into each well of the 96-well plates. The final concentration of the used bacteria was about 6 × 10^5^ cfu/ml. The final concentration of LBD8 peptide was set as 64, 32, 16, 8, 4, 2, 1, 0.5 μM. The MIC assay for modified LBD8 peptides was followed the same procedure. Phosphate buffered saline (PBS) and the same concentration of pGFP peptide were used as blank group and negative controls. Each treatment was performed in four replicates. Then, the mixtures were incubated at 28 or 37°C for 6–8 h depending on different bacterial strains. A precision micro-plate reader (TECAN infinite M200 PRO, Salzburg, Austria) was used to detect the absorbance at 600 and 560 nm for Gram-positive bacteria and Gram-negative bacteria, respectively.

### RNA Interference and Bacterial Infection

RNA interference was carried out to study the *in vivo* function of *FcALF8* gene. A pair of primers with T7 promoter sequence, FcALF8-dsF/FcALF8-dsR (Table 1), were designed to amplify the cDNA fragment of *FcALF8* gene followed the PCR program described below: 94°C for 4 min; 35 cycles of 94°C for 30 s, 61°C for 30 s, and 72°C for 30 s; followed by one cycle of 72°C for 10 min. The PCR product was purified by QIAquick PCR Purification Kit (Qiagen, Germany) and used as the template for dsRNA (dsFcALF8) synthesis under the manufacture's protocols of TranscriptAid T7 High Yield Transcription Kit (Thermo Fisher Scientific, USA). Primers EGFP-dsF and EGFP-dsR with the T7 promoter sequence (Table 1) were designed to amplify a 289 bp DNA fragment of enhanced green fluorescent protein (EGFP) gene from the pEGFPN1 plasmid. The fragment was used for synthesis of exogenous control dsRNA (dsEGFP).

For dosage optimization, dsALF8 was injected into each individual in the dosage of 0.25, 0.50, and 2.00 μg/g shrimp (with an average body length of 6.68 cm and an average body weight of 3.97 g) in the RNAi experiment, respectively. Equal amount of dsEGFP was used to inject shrimp in control groups. Cephalothoraxes of nine individuals were sampled as three biological replicates at 48 h after dsRNA injection. Total RNA extraction and cDNA synthesis were performed following the procedure described in section Total RNA Extraction and cDNA Synthesis. Primers FcALF8-qF and FcALF8-qR (Table 1) were used to detect the expression levels of *FcALF8*. The program for the quantitative RT-PCR was as follows: denaturation at 94°C for 2 min; 40 cycles of 94°C for 20 s, 59°C for 20 s, and 72°C for 20 s. Four technical replicates were set and amplification of 18S rRNA by primers 18S-qF and 18S-qR (Table 1) was performed as an internal reference. The PCR condition was the same except that the annealing temperature was 56°C. The data was analyzed using the 2^−ΔΔCT^ method (30).

After dosage optimization, 0.50 μg/g shrimp was chosen as the experimental dosage. As *V. harveyi* was a common aquatic pathogen and LBD8 peptide showed apparent antibacterial activity against it based on the MIC analysis, *V. harveyi* was used as the experimental pathogen in function analysis. Thirty individuals were equally divided into experiment group (dsALF8+*Vh*) and control group (dsEGFP+*Vh*). Each group contained five biological replicates, with three individuals in each replicate. For dsALF8+*Vh* group, 1 μg dsALF8 was injected into each shrimp. After 48 h, 2 × 10^5^ CFU *V. harveyi* was injected into each shrimp. In the control group, dsEGFP was injected into each shrimp instead of dsALF8. At 27 h post infection, the lymphoid organ, and hepatopancreas of each shrimp was dissected for evaluation of the bacteria count.

### Peptide Injection and Bacterial Infection

Thirty individuals were used for peptide and bacteria injection. They were divided into experiment group (LBD8+*Vh*) and control group (pGFP+*Vh*). Each group contained five biological replicates, with three individuals in each replicate. The synthetic LBD8 peptide, with a final concentration of 64 μM, was mixed with *V. harveyi* and 10 μL of the mixture containing 2 × 10^5^ CFU bacteria was immediately injected into each shrimp in the LBD8+*Vh* group. In the control group, the synthetic pGFP peptide was mixed with *V. harveyi* and injected into each shrimp instead of LBD8 peptide. At 27 h post infection, the lymphoid organ and hepatopancreas of each shrimp was dissected for counting and identification of bacteria.

### Bacterial Count and Strain Identification

The collected lymphoid organ and hepatopancreas samples were weighted, crushed, and blended in sterile PBS separately. Then, 100 μL of the solution was seeded onto TCBS agar media and incubated at 28°C for 18 h. The number of total viable bacteria was counted and 10 single colonies of the dominant bacteria were picked as DNA templates for PCR amplification. Bacteria identification was carried out through sequencing and analyzing a partial sequence of the 16S rDNA that was amplified from the abovementioned DNA templates with the universal primers 27F (31) and 1492R (32).

## Results

### Nucleotide and Deduced Amino Acid Sequences of *FcALF8* Gene

**Figure 1:**
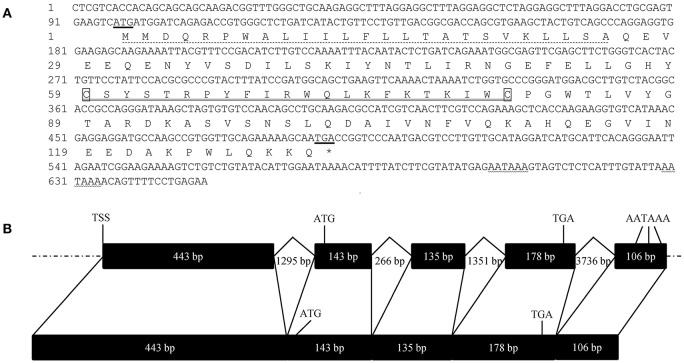
Nucleotide sequence, deduced amino acid sequence and gene structure of *FcALF8*. **(A)** showed the nucleotide sequence and deduced amino acid sequence of *FcALF8*. The start codon and stop codon were underlined. The predicted polyadenylation sites were wave-underlined. The predicted signal peptide was dotted-underlined. The putative LPS-binding domain was double-underlined. **(B)** showed the gene structure of *FcALF8*. Exons and introns with indicated length were shown as black boxes and broken lines, respectively. Flanking DNA sequences were shown as dotted lines. TSS showed the putative **t**ranscription **s**tart **s**ite. The location of start codon (ATG), stop codon (TGA), and polyadenylation signals (AATAAA) were marked in the DNA and mRNA sequences of *FcALF8*.

The full length cDNA of *FcALF8* is 1,005 bp, with an open reading frame (ORF) of 393 bp (MH998632). The genomic DNA sequence is 9230 bp (MH998633). The putative 5′ flanking DNA sequence is 1,577 bp and the *FcALF8* gene contains five exons and four introns (Figure 1A). The start codon and stop codon locate in the second and forth exon, respectively. Three polyadenylation signals (AATAAA) are localized in the fifth exon (Figure 1B). The deduced amino acid sequence consists of 130 residues, with a predicted signal peptide of 25 amino acid residues and a putative LPS-binding domain (LBD) of 22 amino acid residues (Figure 1C).

### FcALF8 Shows Apparent Sequence Difference With Reported ALFs and Specific Tissue Expression Pattern

**Figure 2:**
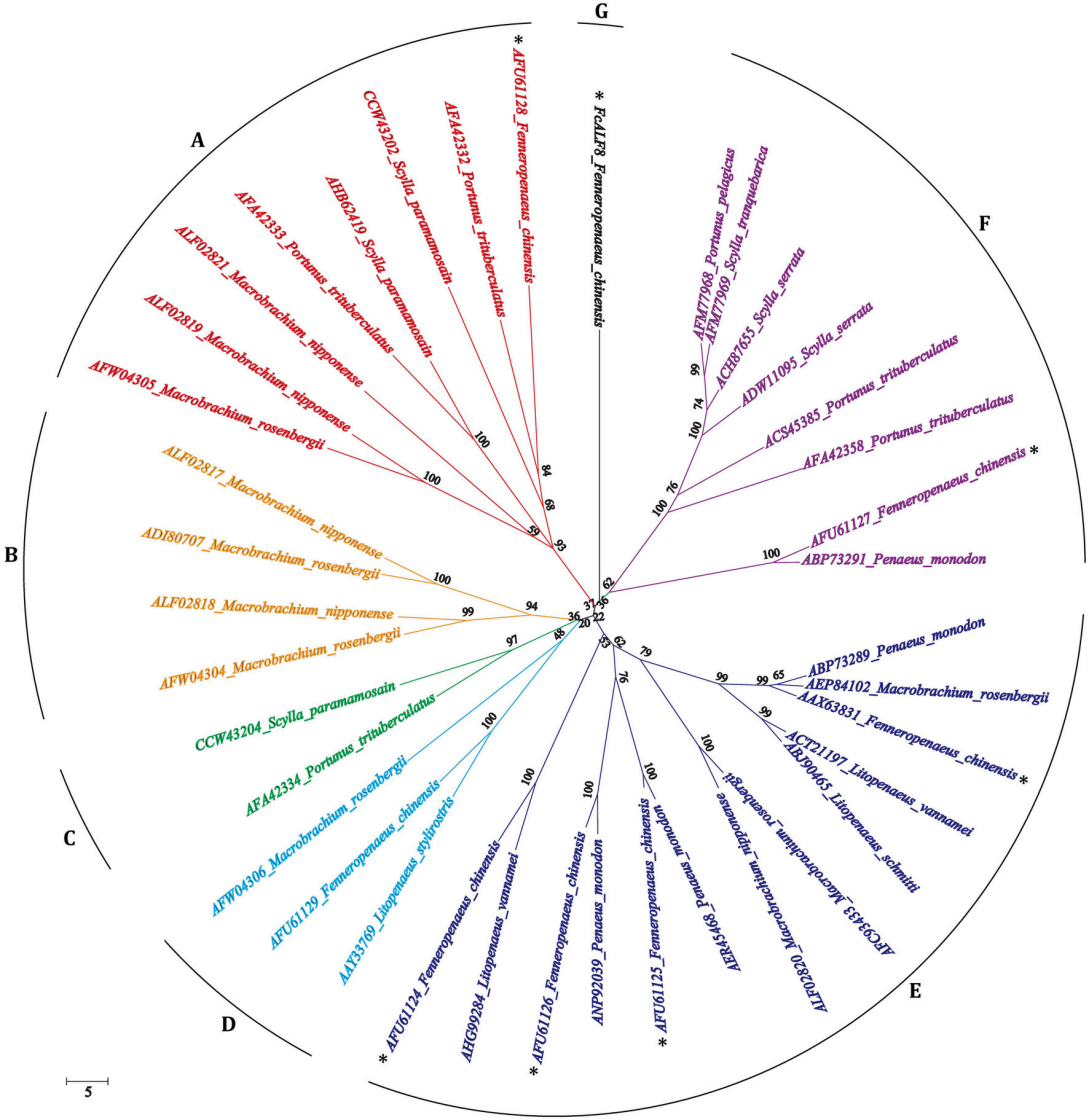
Bootstrapping phylogenetic analyses of anti-lipopolysaccharide factors (ALFs) from twelve crustacean species. The ALFs were showed with GenBank accession numbers listed in Table 2. Whole deduced amino acid sequences excluding signal peptide of ALFs were used for phylogenic analysis by Neighbor-Joining. Bootstrap value was set at 1,000. Percentage of bootstrap replications and divergence distance were shown in the figure. The branches of different type ALFs (with <40% of bootstrap replications) were shown with different colors and capital letters **(A–G)**. ALF sequences from *F. chinensis* were marked with a star (*).

The full length sequences of 39 ALF proteins excluding signal peptides from 12 crustacean species were used for phylogenetic analysis. As shown in Figure 2, all mature ALF proteins are classified into eight categories, among which the bootstrap replications are <40%. Among these ALFs, 38 of them are classified into seven categories, while only FcALF8 is classified into another separate category.

**Table 2:** The information of ALFs used in the present study.

**Species**	**Gene name**	**Accession number**
*Fenneropenaeus chinensis*	Antilipopolysaccharide factor isoform 1	AFU61124
*Fenneropenaeus chinensis*	Antilipopolysaccharide factor isoform 2	AFU61125
*Fenneropenaeus chinensis*	Antilipopolysaccharide factor isoform 3	AFU61126
*Fenneropenaeus chinensis*	Antilipopolysaccharide factor isoform 4	AFU61127
*Fenneropenaeus chinensis*	Antilipopolysaccharide factor isoform 5	AFU61128
*Fenneropenaeus chinensis*	Antilipopolysaccharide factor isoform 6	AFU61129
*Fenneropenaeus chinensis*	Antimicrobial peptide (*FcALF7*)	AAX63831
*Fenneropenaeus chinensis*	Antilipopolysaccharide factor isoform 8	MH998632
*Macrobrachium nipponense*	Anti-lipopolysaccharide factor 5	ALF02821
*Macrobrachium nipponense*	Anti-lipopolysaccharide factor 4	ALF02820
*Macrobrachium nipponense*	Anti-lipopolysaccharide factor 3	ALF02819
*Macrobrachium nipponense*	Anti-lipopolysaccharide factor 1	ALF02818
*Macrobrachium nipponense*	Anti-lipopolysaccharide factor 2	ALF02817
*Portunus trituberculatus*	Anti-lipopolysaccharide factor isoform 7	AFA42358
*Portunus trituberculatus*	Anti-lipopolysaccharide factor isoform 6	AFA42334
*Portunus trituberculatus*	Anti-lipopolysaccharide factor isoform 4	AFA42332
*Portunus trituberculatus*	Anti-lipopolysaccharide factor isoform 5	AFA42333
*Portunus trituberculatus*	Anti-lipopolysaccharide factor isoform 3	ACS45385
*Portunus pelagicus*	Anti-lipopolysaccharide factor precursor	AFM77968
*Scylla serrata*	Anti-lipopolysaccharide factor	ADW11095
*Scylla serrata*	Antilipopolysaccharide factor precursor	ACH87655
*Scylla paramamosain*	Anti-lipopolysaccharide factor	AHB62419
*Scylla paramamosain*	Anti-lipopolysaccharide factor isoform 4	CCW43202
*Scylla paramamosain*	Anti-lipopolysaccharide factor-6	CCW43204
*Scylla tranquebarica*	Anti-lipopolysaccharide factor precursor	AFM77969
*Penaeus monodon*	Anti-lipopolysaccharide factor isoform 3	ABP73289
*Penaeus monodon*	Anti-lipopolysaccharide factor isoform 6	AER45468
*Penaeus monodon*	Anti-lipopolysaccharide factor isoform 2	ABP73291
*Penaeus monodon*	Anti-lipopolysaccharide factor isoform 7	ANP92039
*Litopenaeus vannamei*	Anti-lipopolysaccharide factor isoform 1	AHG99284
*Litopenaeus vannamei*	Anti-lipopolysaccharide factor AV-K isoform	ACT21197
*Litopenaeus schmitti*	Anti-lipopolysaccharide factor	ABJ90465
*Litopenaeus stylirostris*	Anti-lipopolysaccharide factor	AAY33769
*Macrobrachium rosenbergii*	Anti-lipopolysaccharide factor	AFC93433
*Macrobrachium rosenbergii*	Anti-lipopolysaccharide factor	AEP84102
*Macrobrachium rosenbergii*	Anti-lipopolysaccharide factor 3	AFW04306
*Macrobrachium rosenbergii*	Anti-lipopolysaccharide factor 2	AFW04305
*Macrobrachium rosenbergii*	Anti-lipopolysaccharide factor 1	AFW04304
*Macrobrachium rosenbergii*	Anti-lipopolysaccharide factor 2	ADI80707

**Figure 3:**
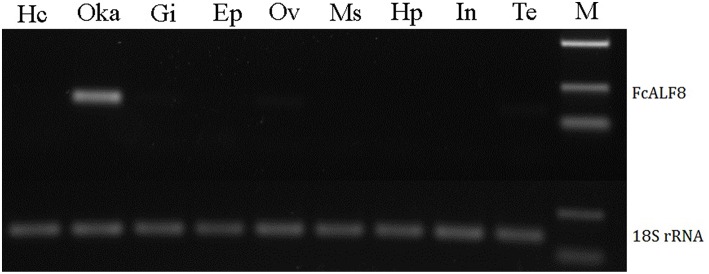
Tissue distribution of FcALF8. Hc, hemocyte; Oka, lymphoid organ; Gi, gill; Ep, epidermis; Ov, ovary; Ms, muscle; Hp, hepatopancreas; In, intestine; Te, testis; M, DL2000 marker. 18S rRNA was used as internal reference gene.

Nine tissues, including hemocytes, lymphoid organ, gill, epidermis, ovary, muscle, hepatopancreas, intestine, and testis were collected for expression analysis of *FcALF8* transcripts. The results showed that *FcALF8* was specifically expressed in the lymphoid organ (Oka, Figure 3).

### Anti-bacterial Activities of LBD8 Peptide

The anti-bacterial activities of LBD8 were confirmed by detecting the minimal inhibitory concentration of synthetic LBD8 peptide on different bacteria. The results showed that LBD8 could inhibit six of the seven tested bacteria (Table 3, column LBD8). LBD8 showed strong activities against *M. luteus* (4–8 μM), *V. alginolyticus* (0.5–1 μM), *V. harveyi* (1–2 μM), and *P. damselae* (1–2 μM). LBD8 also shows activities against *S. epidermidis* (32–64 μM) and *E. coli* (32–64 μM). However, LBD8 shows no activity against *V. parahemolyticus* and *V. campbellii* (>64 μM).

**Table 3:** Minimal inhibitory concentration (μM) of synthetic LBD8 and modified peptides on different bacteria.

**Bacteria**	**LBD8**	**LBD8L**	**LBD8Q**	**LBD8G**
**GRAM-POSITIVE BACTERIA**				
*Micrococcus luteus*	4–8	>64	16–32	4–8
*Staphylococcus epidermidis*	32–64	>64	>64	16–32
**GRAM-NEGATIVE BACTERIA**				
*Vibrio alginolyticus*	0.5–1	>64	8–16	16–32
*Vibrio harveyi*	1–2	>64	2–4	4–8
*Photobacterium damselae*	1–2	>64	4–8	4–8
*Escherichia coli*	32–64	>64	32–64	>64
*Vibrio parahemolyticus*	>64	>64	>64	>64
*Vibrio campbellii*	>64	>64	>64	>64

### *In vivo* Anti-bacterial Function of *FcALF8*

**Figure 4:**
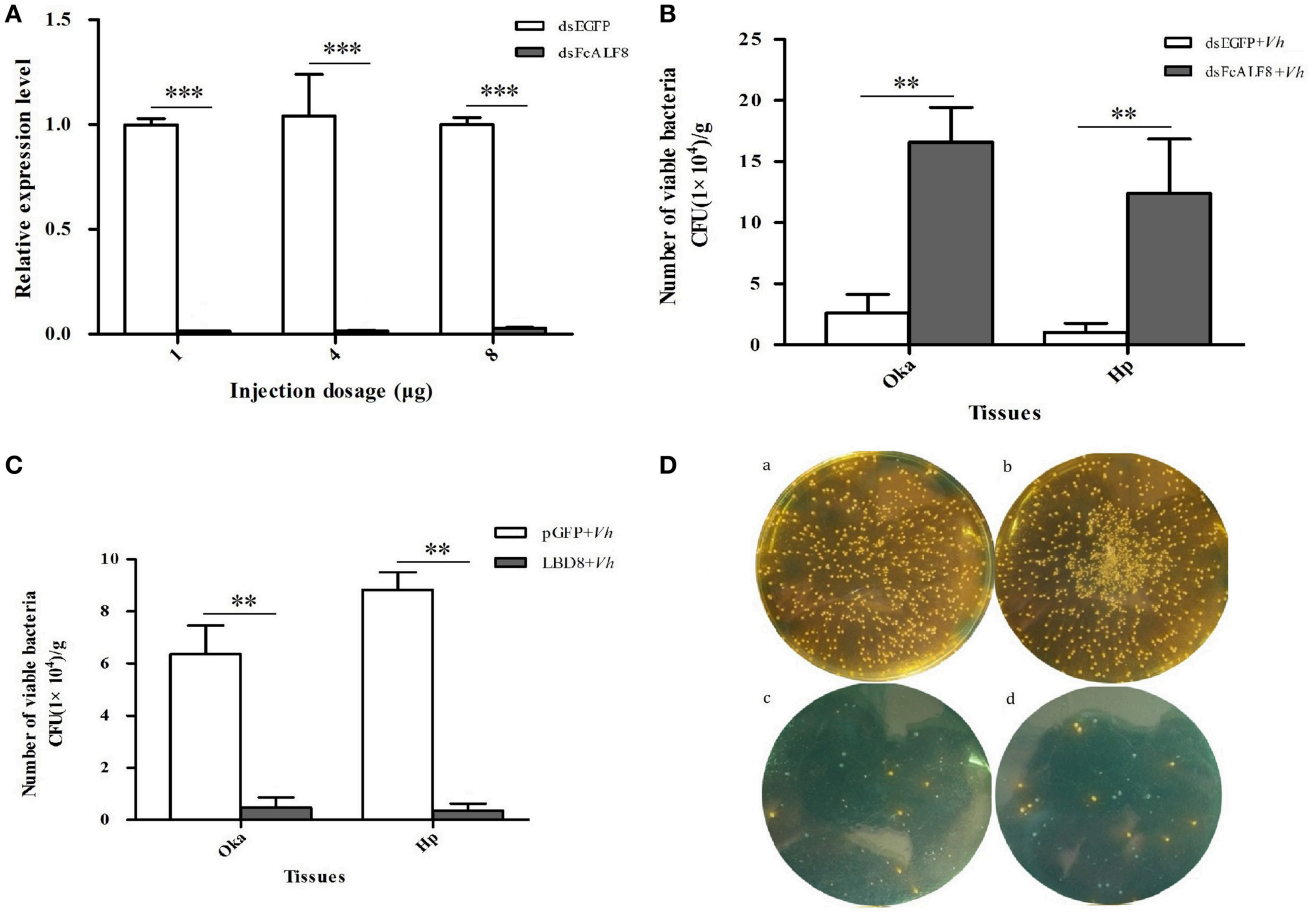
*In vivo* anti-bacterial function of FcALF8 in *F. chinensis*. **(A)** showed the silence efficiency of *FcALF8* after injection of 1, 4, and 8 μg dsRNA. dsEGFP, injected with EGFP dsRNA; dsFcALF8, injected with FcALF8 dsRNA. **(B)** showed total viable bacteria counts in lymphoid organ (Oka) and hepatopancreas (Hp) of shrimp injected with dsRNA and *V. harveyi*. dsEGFP+*Vh*, injected with EGFP dsRNA and *V. harveyi*; dsFcALF8+*Vh*, injected with FcALF8 dsRNA and *V. harveyi*. **(C)** showed total viable bacteria counts in lymphoid organ (Oka) and hepatopancreas (Hp) of shrimp injected with synthetic peptide and *V. harveyi*. pGFP+*Vh*, injected with synthetic pGFP peptide and *V. harveyi*; LBD8+*Vh*, injected with synthetic LBD8 peptide and *V. harveyi*. **(D)** showed spread plates of Oka and hepatopancreas homogenate of shrimp injected with synthetic peptide and *V. harveyi*. (a), Oka homogenate of shrimp injected with pGFP and *V. harveyi*; (b), hepatopancreas homogenate of shrimp injected with pGFP and *V. harveyi*; (c), Oka homogenate of shrimp injected with LBD8, and *V. harveyi*; (d), hepatopancreas homogenate of shrimp injected with LBD8 and *V. harveyi*. Yellow colonies were detected as *V. harveyi*, while green colonies were detected as *Vibrio campbellii*. Significant differences at *P* < 0.01 and *P* < 0.001 between two treatments were shown with two stars (**) and three stars (***), respectively.

In order to study its *in vivo* anti-bacterial function, *FcALF8* was knocked-down by dsRNA mediated RNA interference. All three different dsRNA dosages, including 1, 4, and 8 μg dsRNA per individual, could significantly reduce the expression level of *FcALF8* by 98.6, 98.6, and 97.2%, respectively (Figure 4A). After silenced with 1 μg dsRNA of *FcALF8* and then injected with *V. harveyi*, bacteria proliferation (mainly *V. harveyi*, data not shown) in shrimp is much faster than that in the control group. In the lymphoid organ, the viable bacteria reach 2.22 × 10^5^ CFU/g in *FcALF8* silenced shrimp, which is about 9-folds higher than that in control (2.56 × 10^4^ CFU/g, Figure 4B). In hepatopancreas, the viable bacteria reach 2.03 × 10^5^ CFU/g in *FcALF8* silenced shrimp, which is about 33-fold higher than that in control (6.15 × 10^3^ CFU/g, Figure 4B).

The function of *FcALF8* against *V. harveyi* was further studied through *in vivo* analysis of the anti-bacterial activity of the synthetic LBD8 peptide. Simultaneous injection of LBD8 and *V. harveyi* could inhibit the *in vivo* proliferation of the bacteria. In the LBD8+*Vh* group, the viable bacteria are 4.77 × 10^3^ CFU/g in lymphoid organ and 3.64 × 10^3^ CFU/g in hepatopancreas, which are significantly lower than those in lymphoid organ (6.36 × 10^4^ CFU/g) and hepatopancreas (8.84 × 10^4^ CFU/g) of the control shrimp (Figure 4C). Bacteria identification analysis shows that the predominant bacteria are *V. harveyi* in the control group (yellow spots in Figure 4Da,b). However, the number of *V. harveyi* are very small in LBD8+*Vh* group (yellow spots in Figure 4Dc,d), which is even less than *V. campbellii* (blue spots in Figure 4Dc,d).

### LBDs Show High Sequence Similarity When Considering the Amino Acid Properties

**Figure 5:**
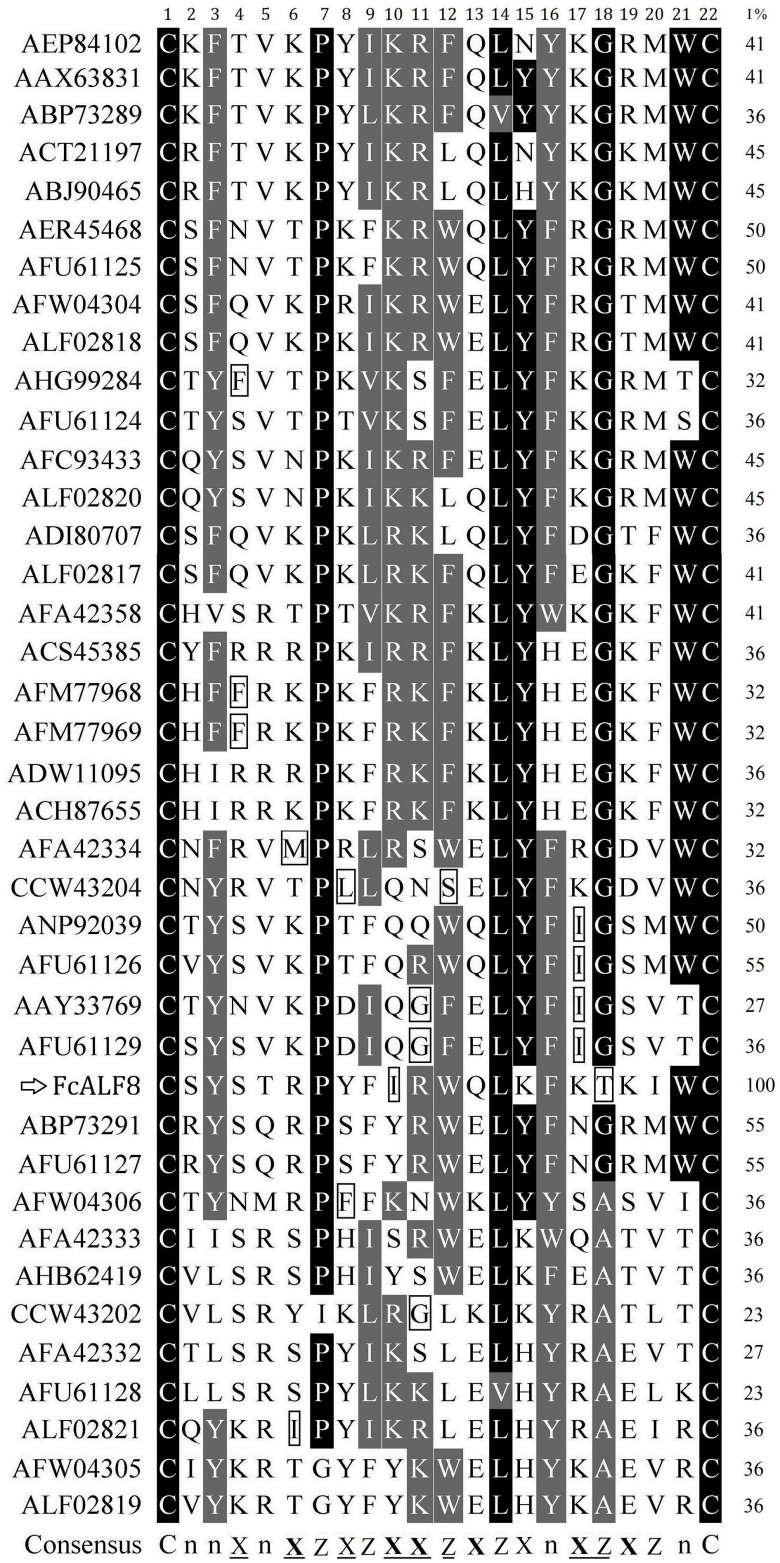
Sequence alignments of LBD peptides from different ALFs. The LBD peptides were showed with GenBank accession numbers of corresponding ALFs listed in Table 2. The amino acid residues in LBD sequences were numbered from 1 to 22. Identical and similar residues were shown in dark and gray, respectively. The % identities between LBD8 and other LBDs were listed in the right column (I%). The consensus sequence was generated by comparing the hydrophilic or hydrophobic properties of the amino acid residues in LBD peptides. In the consensus sequence, X showed hydrophilic residues, Z showed hydrophobic residues, and n showed variable residues. The underlined sites showed existence of <4 residues with distinct property. Six bolded **X** indicated the sites frequent existence of basic or acidic residues among these LBD sequences. Two conserved cysteine residues (C) were shown at two sides of the consensus sequence.

In order to know the relationship between the amino acid sequence of LBD8 and its high antibacterial activity, sequence alignments were performed among LBD8 and other 38 LBD sequences. The result shows that two cysteine residues responsible for disulfide bond formation are conserved while no other residues are identical among these LBDs (Figure 5). However, 17 of the 22 sites in LBD share the similar hydrophilic or hydrophobic properties. Nine sites, including six hydrophobic and three hydrophilic sites are identical among all the LBD sequences. Another eight sites, including six hydrophilic residue preferred sites and two hydrophobic residue preferred sites exist in these LBD sequences. The consensus sequence of the 39 LBD peptides could be described as “CnnXnXZXZXXZXZXnXZXZnC,” where X shows hydrophilic residues, Z shows hydrophobic residues, and n shows variable residues. In the consensus sequence, the underlined sites show the existence of <4 residues with distinct property, that is the identities of these sites are higher than 89.74%. The properties of the 10th (Isoleucine, hydrophobic residue) and 18th (Threonine, hydrophilic residue) residues of the LBD8 are inconsistent with all other LBDs (Figure 5).

### The Structure-Activity Relationship of LBD8 Peptide

In order to study the effects of the disulfide bond and amino acid property of LBD8 on its antibacterial activity, we performed the MIC assay on three modified LBD8 peptides including LBD8L, LBD8Q, and LBD8G. After linearization, the MICs of the peptide on both Gram-positive bacteria and Gram-negative bacteria were all higher than 64 μM (Table 3, column LBD8L), suggesting that LBD8L lost the antibacterial activity. The MIC values of LBD8Q on *M. luteus, S. epidermidis, V. alginolyticus, V. harveyi, P. damselae, E. coli, V. parahemolyticus*, and *V. campbellii* were 16–32, >64, 8–16, 2–4, 4–8, 32–64, >64, and >64 μM, respectively (Table 3, column LBD8Q). Almost all the MIC values of LBD8Q were higher than those of LBD8, except for the MIC on *E. coli*, which was the same. The MIC values of LBD8G on *M. luteus, S. epidermidis, V. alginolyticus, V. harveyi, P. damselae, E. coli, V. parahemolyticus*, and *V. campbellii* were 4–8, 16–32, 16–32, 4–8, 4–8, >64, >64, and >64 μM, respectively (Table 3, column LBD8G). All the MIC values of LBD8G on Gram-negative bacteria were higher than those of LBD8. The data showed that the antibacterial activity of the peptide was apparently changed after the 10th hydrophobic residue Isoleucine was replaced by a hydrophilic residue Glutamine (LBD8Q) or the 18th hydrophilic residue Threonine was replaced by a hydrophobic residue Glycine (LBD8G).

## Discussions

In crustaceans, anti-lipopolysaccharide factors (ALFs) are crucial immune effectors which have a broad spectrum of anti-microbial activities (21). As a type of representative anti-microbial peptides, ALFs are characterized by their diversities. In *F. chinensis*, seven isoforms of ALF genes were identified from different genomic loci, indicating a diversity of ALF genes in shrimp (26). There are also several reported ALFs encoded by different genes in the crab *P. trituberculatus* (13–16). Beside distinct genomic loci, alternative splicing and the presence of SNPs also contribute to the sequence diversity of ALFs (4, 22). The nucleotide sequence and deduced amino acid sequence of *FcALF8* show low similarities with reported *FcALFs*, indicating that it is encoded by a new genomic locus, rather than generated by alternative splicing or presence of SNPs. Based on the genomic organization and amino acid sequence comparisons, ALFs from the penaeid shrimp could be categorized into four groups (33). However, phylogenetic analysis of the whole amino acid sequence of FcALF8 and 38 reported ALFs excluding signal peptide shows that they are divided into seven different groups, groups A to G. FcALF8 is classified into one group alone due to its low sequence similarity with other ALFs, which also indicates that *FcALF8* is a novel ALF gene in *F. chinensis*.

The diversity of ALFs is also evidenced by their tissue distribution patterns as well. In *F. chinensis*, the transcripts of reported *FcALFs* exhibit diverse tissue distribution patterns. *FcALF1* is mainly detected in stomach and lymphoid organ, *FcALF3* is mainly in lymphoid organ and nerve cord, *FcALF4*, and *FcALF5* are mainly in eyestalk and lymphoid organ, *FcALF7* is mainly in lymphoid organ and hemocytes, while *FcALF2* and *FcALF6* are widely distributed in most detected tissues (26). Different from the reported *FcALFs, FcALF8* is specifically expressed in the lymphoid organ. The diversity of ALFs might facilitate their anti-microbial activities against different pathogens in the crustacean innate immune system.

Both *in vitro* and *in vivo* functional studies suggest that FcALF8 has strong activities against several *Vibrio* pathogens. Shrimp suffers infections from various *Vibrio* pathogens, such as *Vibrio alginolyticus* (34), *Vibrio harveyi* (35), *Vibrio parahemolyticus* (36), *Photobacterium damselae* (37), and *Vibrio campbellii* (38). MIC assay shows that although the synthetic LBD8 peptide doesn't show inhibition activity against *V. parahemolyticus* and *V. campbellii*, it exhibits strong inhibition against *V. alginolyticus, V. harveyi*, and *P. damselae*, which are important pathogenic *Vibrio* pathogens in aquaculture. The data indicate that the peptide has obvious potential of drug development for aquaculture. In previous studies, the *in vivo* function of ALF has been investigated through gene knock-down or over-expression analysis. Gene knock-down of ALF will increase the risk of pathogens infection (5, 6), while over-expression of ALF will reduce the risk of pathogen infection in shrimp (9). After knock-down of *FcALF8*, the viable bacteria apparently increased in the lymphoid organ and hepatopancreas of shrimp after *V. harveyi* infection. On the contrary, injection of pre-incubated *V. harveyi* with LBD8 into shrimp significantly reduced its proliferation in the lymphoid organ and hepatopancreas. These data suggest that FcALF8 has great anti-bacterial activity to *V. harveyi*, which is consistent with the MIC assay.

It was also notable that few bacteria colonies were grown in the medium of LBD8+*Vh* group. However, the majority in these few bacteria are *V. campbellii* rather than *V. harveyi*. The phenomenon could be explained from two aspects. On one hand, *V. campbellii* might also exist in the control group (pGFP+*Vh* group), while they were concealed by the mass of *V. harveyi*. On the other hand, the *in vivo* flora balance might be broken after LBD8 injection and LBD8 has no activity to *V. campbellii*, which is also confirmed by MIC assay. In *Exopalaemon carinicauda*, knock-down of *EcALF1* also causes *in vivo Vibrio* proliferation and even a lesion of hepatopancreas and individual death (7). The evidence suggests that ALF plays important roles in host immune system by maintaining *in vivo* flora balance.

As the functional domain of ALF, LPS-binding domain (LBD) is characterized by two conserved cysteine residues at both ends of the domain (8). The two cysteine residues determine whether LBDs have antibacterial activity by forming a disulfide bond, because the antibacterial activity of LBD8 is lost after linearization. However, the amino acid sequence similarity of LBDs is also very low. Amino acid sequences alignment shows that only two cysteine residues are conserved in all LBDs. There are only three identical sites in LBDs even in the seven previously reported FcALFs (26). However, the identical sites are dramatically increased when considering the property of the amino acid residues in LBDs. When the properties of the amino acids are classified into hydrophobic, neutral, basic, and acidic amino acids, there are eight identical sites in LBDs of the seven previously reported FcALFs (26). When the properties of the amino acids are classified into hydrophobic and hydrophilic amino acids, 17 of the 22 residue sites, including nine identical sites and eight highly similar sites, exist in all 39 analyzed LBDs. It is notable that the amino acid properties at the position 10 and 18 of all other LBD peptides are conserved, which is hydrophilic at position 10 and is hydrophobic at position 18. However, the amino acid properties at these two positions of LBD8 are totally different, which is hydrophobic at position 10 and is hydrophilic at position 18. The difference of the amino acid property at the two sites might partially cause LBD8 to be a new group. Besides, this might also contribute to higher antibacterial activities of LBD8 than those of other LBD peptides from the Chinese shrimp (26). A further activity analysis on modified LBD8 peptides shows that the antibacterial activity of LBD8 is apparently reduced after changing the amino acid property at position 10 or 18. These data suggest that the disulfide bond and amino acid property contributes to the conservation of the functional LBDs. In addition, the finding provides new insights in the design of antimicrobial peptides.

Most ALF belongs to cationic antimicrobial peptides (AMPs), in which the cationic property owing to basic amino acids is considered as an important factor for binding to negatively charged surface in lipid membrane of bacteria (39, 40). The net charge of cationic α-helical AMPs greatly modulates the antimicrobial specificity and efficacy of these peptides (41). Even if belonging to β-sheet, the basic amino acids and net charge in LBD peptides are also important for their antimicrobial activities, and the absence of basic amino acids in LBD peptides leads to loss of antimicrobial functions in *F. chinensis* (42). A total of five basic amino acids, including two arginine residues and three lysine residues, and none acidic amino acids exist in the LBD peptide of FcALF8. MIC assay indicates that the LBD of FcALF8 shows strong anti-bacterial activities, especially to gram negative bacteria. Strong anti-bacterial activities are also found from the LBD peptides of FcALF2 and ALFFc, which contain five or six basic amino acids and zero acidic amino acids, respectively. However, other LBD peptides with less number of basic amino acids and one or two acidic amino acids show relatively lower anti-bacterial activities to tested bacteria (26). A modified LBD peptide, LBDv, which is derived from the LBD peptide of FcALF2 by changing five polar amino acids into basic amino acids, presents stronger activities than the LBD peptide of FcALF2 (43). These data support the opinion that basic amino acids and net charge are important to the activity of LBDs.

In conclusion, a novel *ALF* gene, *FcALF8*, specifically expressed in the lymphoid organ, was identified from *F. chinensi*s. The LBD peptide of FcALF8 shows strong activities to the gram negative bacteria *V. alginolyticus, V. harveyi* and *Photobacterium damselae* and the gram positive bacteria *Micrococcus luteus. In vivo* study confirms its antibacterial activity to *V. harveyi* and further indicates that ALF might function in host immune system by maintaining *in vivo* flora balance. Sequence analysis of LBDs suggests that the amino acid property, especially the basic amino acids and net charge, largely contributes to the conservation of the functional domain. The present data provide new insights into ALF functions in crustaceans and will give useful guidance for the design of LBD-originated antimicrobial agents.

## Author Contributions

SL, FL, and JX conceived and designed the project. SL and XL performed all the experiments and prepared the figures. SL and FL wrote the manuscript. All authors reviewed the manuscript.

### Conflict of Interest Statement

The authors declare that the research was conducted in the absence of any commercial or financial relationships that could be construed as a potential conflict of interest.
